# Analysis of the correlation between blood glucose level and prognosis in patients younger than 18 years of age who had head trauma

**DOI:** 10.1186/s13017-015-0010-0

**Published:** 2015-02-24

**Authors:** Bahadir Danisman, Muhittin Serkan Yilmaz, Bahattin Isik, Cemil Kavalci, Cihat Yel, Alper Gorkem Solakoglu, Burak Demirci, Selim Inan, M Evvah Karakilic

**Affiliations:** Emergency Department, Dıskapi Yıldırım Beyazit Training and Research Hospital, Ankara, Turkey; Emergency Department, Numune Training and Research Hospital, Ankara, Turkey; Emergency Department, Baskent University Faculty of Medicine, Ankara, Turkey

**Keywords:** Head trauma, Blood glucose, GCS

## Abstract

**Objective:**

To analyze the correlation between early-term blood glucose level and prognosis in patients with isolated head trauma.

**Methods:**

This study included a total of 100 patients younger than 18 years of age who had isolated head trauma. The admission blood glucose levels of these patients were measured. Age at the time of the incident, sex, mode of occurrence of the trauma, computed tomography findings, and GCSs were recorded. Kruskall Wallis test was used compare of groups. A p value less than 0.05 was considered statistically significant.

**Results:**

The median age of the study population was 7 years and the median GCS was 11. There was a significant negative correlation between blood glucose level and GCS (p < 0.05). A significant correlation in the negative direction was observed between GCS and blood glucose level (r = −0.658, p < 0.05). Seventy-seven percent of the patients were admitted to hospital, while 6% died in ED.

**Conclusion:**

The results of the present study suggest that hyperglycemia at an early stage and a low GCS may be reliable predictors of the severity of head trauma and prognosis. A higher blood glucose level may be an ominous sign that predicts a poor prognosis and an increased risk of death.

## Introduction

Head trauma is common after general body trauma and requires a multidisciplinary approach. It is a severe health problem with long-term rehabilitation of sequel [1]. With an annual mortality rate of about 200/100000, it ranks third among the most common causes of mortality and morbidity in children [2]. The mortality rate of head trauma may goes up to 20-35% [3,4].

Mortality and morbidity of head trauma may be due to the direct injury produced by the trauma itself on the one side, and due to ischemia and hypoxia secondary to head trauma on the other [2]. These effects include traumatic ischemia, infarction, cardiorespiratory dysfunction, secondary hemorrhages, diffuse cerebral edema, and hypoxic ischemia mediated by neuromediators released by body in response to trauma [4]. In addition, it should also be kept in mind that brain injury secondary to head trauma may lead to impaired systemic hemostasis and organ dysfunction.

Blood glucose possesses all properties of an ideal serum marker of systemic injury. It is highly sensitive for cerebral cellular injury secondary to head trauma. Although the pathophysiology of hyperglycemia’s neuropathic effect is not entirely clear, it has been reported that it aggravates ischemic acidosis, which in turn worsens brain edema [5,6]. Cochran reported that blood glucose levels more than 300 mg/dl is associated with dead [5].

This study aimed to assess the relationship between early-term blood glucose level and prognosis in patients younger than 18 years of age who were admitted to our emergency department after head trauma.

## Materials and method

This study was approved by the local ethics committee and performed prospectively at Dışkapı Yıldırım Beyazıt Training and Research Hospital, Department of Emergency Medicine between 01.01.2010-01.01.2011. Written informed consent was obtained from the patient’s guardian/parent/next of kin for the publication of this report and any accompanying images. It included 100 patients younger than 18 years of age who presented with isolated head trauma and who met the study inclusion criteria (Table 1).

**Table 1:** **İnclusion/exclusion criteria**

**İnclusion criteria**	**Exclusion criteria**
1- < 18 year old	1- > 18 year old
2-isolated head trauma	2-Multiple trauma
3- Admitted within 3 hours after trauma	3- Admitted 3 hours after trauma
	4-Admitted exitus patient
	5- Patients with a GCS of 15 and normal CT findings

Age at the time of the incident, sex, Glasgow Coma Scale (GCS) at the time of admission, findings of computed tomography (CT), trauma mechanism, and blood glucose levels were recorded.

The study data were analyzed using the SPSS 13.00 for Windows software package. The descriptive statistics included percentage, number, and median values. Data distribution was tested with the Kolmogorov Smirnov test. Inter-group comparisons were carried out using the Kruskall Wallis test. A p value less than 0.05 was considered statistically significant.

## Results

The median age of the study population was 7 [2-16] years, with 67% of the study population being male (Table 2). The most common causes of isolated head trauma were out-of-vehicle traffic accident (37%) and falls (30%). The median GCS level was 11 [3-15] (Table 3). Head trauma was severe in 23 patients, moderate in 29, and mild in 48. CT examination most commonly demonstrated combined findings (52%), with brain edema being the most common isolated finding (25%). Seventeen percent of the cases were discharged after emergency care while 77% of them were hospitalized and 6% died during ED stay (Table 4).

**Table 2:** **Distribution of patients characteristics according to groups**

**Variable**		**Median (Min-Max)/n (%)**
Age		7 (2-16)
Sex	Male	67 (67)
	Female	33 (33)
Trauma mechanism	Pedestrian	37 (37)
	Motor vehicle accident	26 (26)
	Falls	30 (30)
	Assault	7 (7)
GCS	Severe head trauma (GCS:3-8)	23 (23)
	Moderate head trauma (GCS:9-13)	29 (29)
	Mild head trauma (GCS:14-15)	48 (48)
CT findings	Brain edema	25 (25)
	Hematoma	14 (14)
	Linear fracture	6 (6)
	Contusion	3 (%3)
	Combined CT findings	52 (%52)
Outcome of patients	Discharge	17 (%17)
	Hospitalization	77 (%77)
	Exitus	6 (%6)

*n:total number of the patient (Combined CT findings: Linear fracture and brain edema, linear fracture and epidural hematoma, etc…).*

*GCS: Glasgow Coma Scale.*

**Table 3:** **The relationship between trauma severity and hyperglycemia**

	**n**	**Glucose (Median (min;max))**	**p**
Severe head trauma	23	177 (98-324)	p<0.05
Moderate head trauma	29	138 (92-244)	
Mild head trauma	48	115 (68-180)

**Table 4:** **The relationship between patient results and hyperglycemia**

	**N**	**Glucose (Median (min;max))**	**p**
Discharge	17	103 (68-145)	p<0.05
Hospitalization	77	118 (86-154)	
Exitus	6	233 (148-324)	

The median blood glucose level was 177 mg/dl in 23 patients with severe head trauma. The median blood glucose levels by trauma severity were summarized on Table 3. Different trauma severities significantly differed with respect to blood glucose levels. A significant negative correlation was detected between GCS and blood glucose level (r = −0.658, p < 0.05).

## Discussion

Head trauma is the most common type of pediatric traumas, and it is considered the leading cause of death between 1–15 years of age [7-10]. The acute stage of severe head trauma is generally characterized by a systemic stress response [10].

Many studies have reported that traffic accidents are the main cause of childhood head traumas [11-13]. Kavalci et al. [14,15] and Durdu et al. [16] reported that the most common trauma mechanism was motor vehicle accident. Our study also found that out-of-vehicle traffic accident was the most common cause of head trauma. Traffic accidents lead to a more destructive process since they apply high energies on the victims.

Murgio et al. reported that 56.4% of the patients had mild head trauma, 38.9% had moderate head trauma, and 4.7% had severe head trauma [17]. Işık et al. reported that 74% of traumas were mild, 22% were moderate, and 4% were severe [8]. A majority of our cases similarly had mild head trauma. However, the rate of severe head trauma in our study was also higher than previous studies. We suggest that this difference may be due to injury by high-energy accidents.

Rovlias et al. reported that patients with head trauma whose blood glucose level was more than 200 mg/dl within 24 hours had worse prognosis [18]. The authors linked this finding to a series of reactions including hyperglycemia following cerebral ischemia [18]. It has also been demonstrated that hyperglycemia in the course of traumatic brain injury adversely affects oxygenation [19]. It has been advocated that, in addition to severity of trauma itself, biochemical and vital parameters should also be taken into account when predicting the severity of head trauma [20]. GCS provides clinicians with very valuable information for predicting head trauma severity. Nevertheless, GCS may also fail in many circumstances. It should be remembered that blood glucose level and vital signs at the acute stage may be good predictors of patient outcomes when GCS is considered insufficient. Furthermore, it looks promising to predict future neurological injury with the help of high blood glucose levels and poor vital signs in patients with normal imaging tests such as CT.

Rovlias et al. [18], in a study with 267 patients with head trauma and a GCS of 3–13, demonstrated that clinical course was worse in patients with a blood glucose level greater than 200 mg/dl. Chiaretti et al. [20] reported that 87.5% of cases with head trauma and a GCS below 8 had a high blood glucose level. Merguerian et al. [21] found a mean blood glucose level of 270 mg/dl in 19 deeply comatose patients with severe head trauma who had a GCS of 3. They also found that all patients with hyperglycemia and a low GCS were lost whereas patients with a low GCS but no hyperglycemia had a mortality rate of 17% on follow-up. Rengachary et al. suggested that blood glucose level elevation was a more potent predictor of clinical worsening than vital signs [22]. Although blood glucose level was closely related to cerebral injury in cases with a GCS of 8 or lower, it was not correlated to the severity of extracranial trauma [23]. Moreover, Babbitt et al. [24] showed that hyperglycemia was related to intracranial injury in younger children. Our study results revealed that the median glucose level was 177 mg/dl in the severe head trauma group and 233 mg/dl in the patients who died. In addition, there was a negative correlation between blood glucose level and GCS. That is, GCS dropped as blood glucose level increased. Our blood glucose levels have been lower than those reported in previous studies since we drew blood samples within three hours of admission. In agreement with the literature, our study demonstrated a parallel worsening in brain injury with elevating blood glucose level. Hyperglycemia may increase mortality by augmenting brain edema and hypoxia.

Movery et al. reported that blood glucose level increased in proportional to trauma severity. Nevertheless, the authors stated glucose level was not the sole determinant of mortality in clinical practice [25]. Cochran et al. found a significantly higher glucose level in patients with an elevated mortality rate and added that hyperglycemia was closely related to mortality [5]. Similar to Cochran’s results, our study demonstrated a significant correlation between mortality and hyperglycemia. This result suggests that hyperglycemia may be an important marker for predicting mortality risk.

## Conclusion

In conclusion, the results of this study supports the notion that in patients with head trauma and a low GCS hyperglycemia at an early stage after head trauma may be a reliable marker of cerebral injury and patient prognosis. An elevated blood glucose level may suggest that a patient’s prognosis is likely poor and the risk of dying is substantially high.
